# Using multivariable Mendelian randomization to estimate the causal effect of bone mineral density on osteoarthritis risk, independently of body mass index

**DOI:** 10.1093/ije/dyab251

**Published:** 2021-12-13

**Authors:** April Hartley, Eleanor Sanderson, Raquel Granell, Lavinia Paternoster, Jie Zheng, George Davey Smith, Lorraine Southam, Konstantinos Hatzikotoulas, Cindy G Boer, Joyce van Meurs, Eleftheria Zeggini, The Genetics of Osteoarthritis Consortium, Lilja Stefánsdóttir, Yanfei Zhang, Rodrigo Coutinho de Almeida, Tian T Wu, Jie Zheng, Maris Teder-Laving, Anne-Heidi Skogholt, Chikashi Terao, Eleni Zengini, George Alexiadis, Andrei Barysenka, Gyda Bjornsdottir, Maiken E Gabrielsen, Arthur Gilly, Thorvaldur Ingvarsson, Marianne B Johnsen, Helgi Jonsson, Margreet G Kloppenburg, Almut Luetge, Reedik Mägi, Massimo Mangino, Rob R G H H Nelissen, Manu Shivakumar, Julia Steinberg, Hiroshi Takuwa, Laurent Thomas, Margo Tuerlings, George Babis, Jason Pui Yin Cheung, Dino Samartzis, Steve A Lietman, P Eline Slagboom, Kari Stefansson, André G Uitterlinden, Bendik Winsvold, John-Anker Zwart, Pak Chung Sham, Gudmar Thorleifsson, Tom R Gaunt, Andrew P Morris, Ana M Valdes, Aspasia Tsezou, Kathryn S E Cheah, Shiro Ikegawa, Kristian Hveem, Tõnu Esko, J Mark Wilkinson, Ingrid Meulenbelt, Ming Ta Michael Lee, Unnur Styrkársdóttir, Celia L Gregson, Jon H Tobias

**Affiliations:** MRC Integrative Epidemiology Unit, Population Health Sciences, Bristol Medical School, University of Bristol, Bristol, UK; Musculoskeletal Research Unit, Translational Health Sciences, Bristol Medical School, University of Bristol, Bristol, UK; MRC Integrative Epidemiology Unit, Population Health Sciences, Bristol Medical School, University of Bristol, Bristol, UK; MRC Integrative Epidemiology Unit, Population Health Sciences, Bristol Medical School, University of Bristol, Bristol, UK; MRC Integrative Epidemiology Unit, Population Health Sciences, Bristol Medical School, University of Bristol, Bristol, UK; MRC Integrative Epidemiology Unit, Population Health Sciences, Bristol Medical School, University of Bristol, Bristol, UK; MRC Integrative Epidemiology Unit, Population Health Sciences, Bristol Medical School, University of Bristol, Bristol, UK; Institute of Translational Genomics, Helmholtz Zentrum München, German Research Center for Environmental Health, 85764, Neuherberg, Germany; Institute of Translational Genomics, Helmholtz Zentrum München, German Research Center for Environmental Health, 85764, Neuherberg, Germany; Department of Internal Medicine and Epidemiology, Erasmus MC, Rotterdam, The Netherlands; Department of Internal Medicine and Epidemiology, Erasmus MC, Rotterdam, The Netherlands; Institute of Translational Genomics, Helmholtz Zentrum München, German Research Center for Environmental Health, 85764, Neuherberg, Germany; Musculoskeletal Research Unit, Translational Health Sciences, Bristol Medical School, University of Bristol, Bristol, UK; MRC Integrative Epidemiology Unit, Population Health Sciences, Bristol Medical School, University of Bristol, Bristol, UK; Musculoskeletal Research Unit, Translational Health Sciences, Bristol Medical School, University of Bristol, Bristol, UK

**Keywords:** Osteoarthritis, bone mineral density, Mendelian randomization, body mass index, UK Biobank

## Abstract

**Objectives:**

Observational analyses suggest that high bone mineral density (BMD) is a risk factor for osteoarthritis (OA); it is unclear whether this represents a causal effect or shared aetiology and whether these relationships are body mass index (BMI)-independent. We performed bidirectional Mendelian randomization (MR) to uncover the causal pathways between BMD, BMI and OA.

**Methods:**

One-sample (1S)MR estimates were generated by two-stage least-squares regression. Unweighted allele scores instrumented each exposure*.* Two-sample (2S)MR estimates were generated using inverse-variance weighted random-effects meta-analysis. Multivariable MR (MVMR), including BMD and BMI instruments in the same model, determined the BMI-independent causal pathway from BMD to OA. Latent causal variable (LCV) analysis, using weight-adjusted femoral neck (FN)–BMD and hip/knee OA summary statistics, determined whether genetic correlation explained the causal effect of BMD on OA.

**Results:**

1SMR provided strong evidence for a causal effect of BMD estimated from heel ultrasound (eBMD) on hip and knee OA {odds ratio [OR]_hip_ = 1.28 [95% confidence interval (CI) = 1.05, 1.57], *p* = 0.02, OR_knee_ = 1.40 [95% CI = 1.20, 1.63], *p* = 3 × 10^–5^, OR per standard deviation [SD] increase}. 2SMR effect sizes were consistent in direction. Results suggested that the causal pathways between eBMD and OA were bidirectional (β_hip_ = 1.10 [95% CI = 0.36, 1.84], *p* = 0.003, β_knee_ = 4.16 [95% CI = 2.74, 5.57], *p* = 8 × 10^–9^, β = SD increase per doubling in risk). MVMR identified a BMI-independent causal pathway between eBMD and hip/knee OA. LCV suggested that genetic correlation (i.e. shared genetic aetiology) did not fully explain the causal effects of BMD on hip/knee OA.

**Conclusions:**

These results provide evidence for a BMI-independent causal effect of eBMD on OA. Despite evidence of bidirectional effects, the effect of BMD on OA did not appear to be fully explained by shared genetic aetiology, suggesting a direct action of bone on joint deterioration.

Key Messages

- Mendelian randomization (MR) analyses suggest that bone mineral density (BMD), assessed from heel ultrasound scans, is a risk factor for osteoarthritis, independently of adiposity.
- Evidence for reverse causality (i.e. a causal effect of osteoarthritis on BMD) may reflect the shared biological pathways contributing to bone and joint development.
- Latent causal variable (LCV) analysis provides evidence for a direct causal effect of BMD on osteoarthritis, which is not fully explained by genetic correlation between these two traits.
- This paper illustrates the utility of methods such as LCV analysis and multivariable MR when examining causal pathways in situations in which complex relationships exist, such as those between BMD, body mass index and osteoarthritis.

## Introduction

Although osteoarthritis (OA) is a major cause of morbidity worldwide, effective pharmacological treatment remains elusive. It may be possible to develop novel therapeutic approaches based on understanding of risk factors. Several large population-based studies have identified positive relationships between bone mineral density (BMD) and hip and knee OA. ^1^ Mendelian randomization (MR), which is commonly used to explore causal relationships,^2–4^ has recently obtained evidence for a causal role of BMD on hip and knee OA risk.^5^ Body mass index (BMI), a risk factor for OA^6–8^ and positively associated with BMD,^9^ may bias MR estimates for the relationship between BMD and OA. Funck-Brentano *et al.* addressed this by excluding instrument(s) associated with BMI.^5^ An alternative approach, yet to be applied in this context, is the use of multivariable MR (MVMR) to estimate the direct causal effect of the exposure on the outcome when the instrument(s) are associated with multiple risk factors.^10^ Alternatively, rather than a causal effect of BMD on OA, shared biological pathways may contribute to both traits. Consistently with this possibility, a genetic correlation between lumbar spine (LS)–BMD and OA has been observed.^11^ Genetic correlation may give rise to bidirectional causal relationships in MR analysis.

As well as the relationship between BMD and OA, relationships with BMI could be characterized by bidirectional relationships. A causal effect of BMI on BMD is well established; the skeleton adapts to the increased load placed upon it by increasing BMD. Alternatively, a causal pathway between BMD and BMI is plausible via the metabolic effects of bone turnover. Murine osteocalcin knockouts have increased fat mass and are insulin-resistant;^12^ in humans, higher BMD is associated with lower circulating osteocalcin, which may mediate the positive association between BMD and fat mass. However, an MR analysis found no evidence of a causal pathway between femoral neck (FN) or LS–BMD and BMI in children.^9^

**Figure 1:**
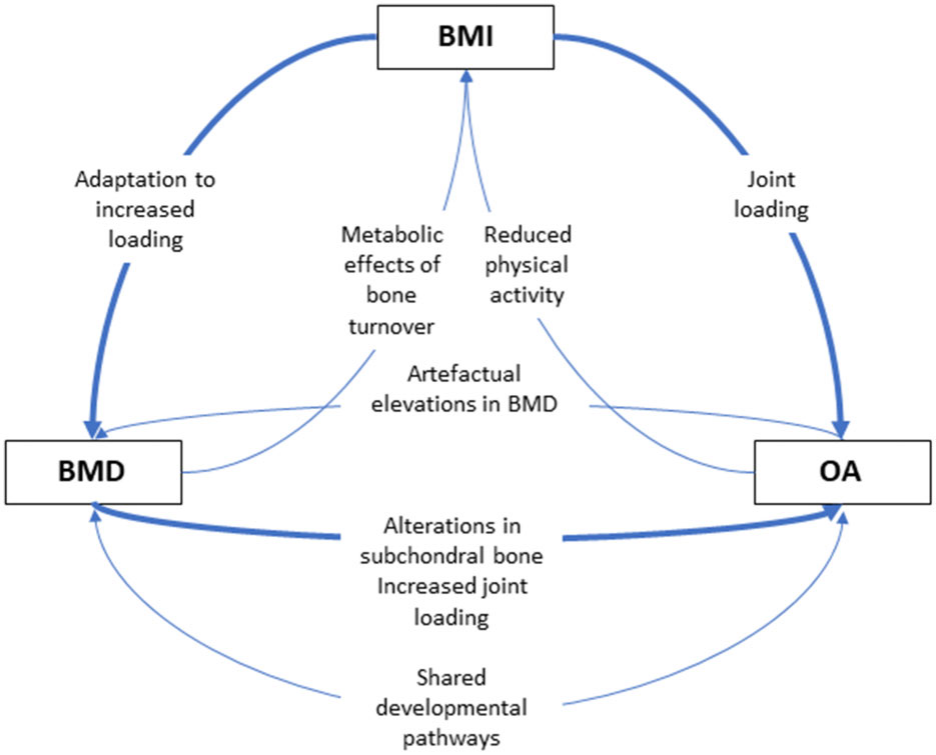
Diagram summarizing hypothesized relationships between bone mineral density, body mass index and osteoarthritis Thicker arrows represent stronger hypothesized relationships. Diagram does not take account of temporality of relationships due to the uncertainty in the temporal sequence, e.g. OA may first cause an increase in BMI due to reduced PA, leading to further OA through greater joint loading; however, it is equally possible that BMI leading to an increase in joint loading is the initiating event. BMD, bone mineral density; BMI, body mass index; OA, osteoarthritis.

To provide a more complete understanding of the relationship between BMD and OA, we tested bidirectional relationships between BMD, OA and BMI (Figure 1) using one-sample (1S) and two-sample (2S) MR, and aimed to determine the direct (i.e. unconfounded) causal pathways between these variables using MVMR.

## Methods

### Individual-level analyses

Individual-level analyses were performed in the UK Biobank population. UK Biobank is a UK-wide population-based health research resource consisting of ∼500 000 people, aged 38–73 years, who were recruited in 2006–2010.^13^ Participants provided a range of information [e.g. demographics, health status, lifestyle/physical activity (PA) measures] via questionnaires and interviews; anthropometric measures and blood samples were taken (data available at www.ukbiobank.ac.uk). A full description of the study design, participants and quality-control methods has been published.^13^ Methods for assessing BMD and ascertaining hospital-diagnosed OA status are described in the Supplementary Information (available as Supplementary data at *IJE* online). UK Biobank received ethical approval from the Research Ethics Committee (REC reference: 11/NW/0382).

Data collection, genotyping, and imputation and observational analyses in UK Biobank are described in the Supplementary Material (available as Supplementary data at *IJE* online).

### MR

**Figure 2:**
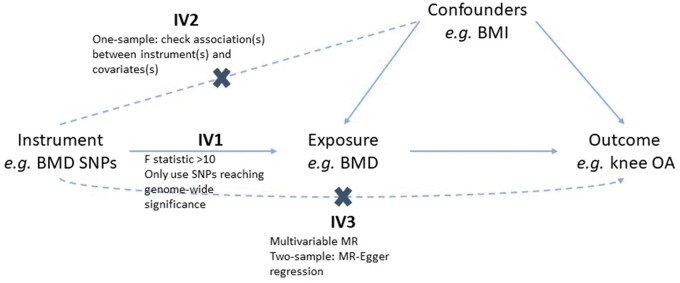
Assumptions of Mendelian randomization and how we tested these assumptions For a Mendelian randomization (MR) effect estimate to be valid, the instrument(s) must satisfy three key assumptions:^3^ IV1 [the instrument(s) must be robustly associated with the exposure], IV2 [the instrument(s) must not be associated with any confounders of the exposure–outcome relationship] and IV3 [the instruments(s) can only be associated with the outcome via the exposure and not via a different biological pathway independent of the exposure (i.e. horizontal pleiotropy)]. In one-sample analyses, IV1 was tested by calculating the F-statistic, which is a measure of instrument strength. A >10 threshold is used to determine sufficient instrument strength.^2^ IV2 was tested by determining the association between the instruments and potential confounders of the exposure–outcome relationship. In two-sample analyses, to satisfy IV1, we ensured that all instruments were robustly associated with the exposure by only including SNPs associated with the exposure at genome-wide significance. To address IV3, MR–Egger regression was performed to generate an estimate of horizontal pleiotropy (intercept) and a pleiotropy-robust estimate of the causal effect (slope). Weighted median regression was performed to determine the robustness of IVW estimates as weighted median estimates are valid even if ≤50% of the SNPs are not valid instruments.^20^ BMD, bone mineral density; BMI, body mass index; OA, osteoarthritis; SNP, single-nucleotide polymorphism.

A summary of all MR analyses performed, along with the source of each of the instruments, is presented in Table 1 and of the assumptions of MR and how we tested these in Figure 2. We examined causal relationships with hip and knee OA separately, given the availability of separate genome-wide association studies (GWAS) for these outcomes, which have no overlap in terms of the most strongly associated single-nucleotide polymorphisms (SNPs).

**Table 1:** Summary of all one-sample and two-sample Mendelian randomization analyses performed

	Exposure		Outcome	
		Source		Source
1S	eBMD	Individual-level eBMD in UK Biobank I: FN–BMD SNPs from GEFOS^21^	Knee OA	Individual-level HD knee OA status in UK Biobank
2S	eBMD	Summary statistics from GEFOS UK Biobank eBMD GWAS *N* = 426 824^46^	Knee OA	Summary statistics from GO consortium GWAS based on radiographic, clinical evaluation, joint replacement, self-reported or HD knee OA, excluding UK Biobank *N* = 44 001 ca, 301 541 co^47^
1S	eBMD	Individual-level eBMD in UK Biobank I: FN–BMD SNPs from GEFOS^21^	Hip OA	Individual-level HD hip OA status in UK Biobank
2S	eBMD	Summary statistics from GEFOS UK Biobank eBMD GWAS *N* = 426 824^46^	Hip OA	Summary statistics from GO consortium GWAS based on radiographic, clinical evaluation, joint replacement, self-reported or HD hip OA, excluding UK Biobank *N* = 25 237 ca, 272 284 co^47^
1S	eBMD	Individual-level eBMD in UK Biobank I: FN–BMD SNPs from GEFOS^21^	BMI	Individual-level BMI data in UK Biobank
2S	eBMD	Summary statistics from GEFOS UK Biobank eBMD GWAS *N* = 426 824^46^	BMI	Summary statistics from GIANT European BMI GWAS *N* = 339 224^48^
1S	BMI	Individual-level BMI data in UK Biobank I: BMI SNPs from GIANT^48^	Knee OA	Individual-level HD knee OA status in UK Biobank
2S	BMI	Summary statistics from GIANT European BMI GWAS *N* = 339 224^48^	Knee OA	Summary statistics from UK Biobank and arcOGEN GWAS HD knee OA *N* = 24 955 ca, 378 169 co^49^
1S	BMI	Individual-level BMI data in UK Biobank I: BMI SNPs from GIANT^48^	Hip OA	Individual-level HD hip OA status in UK Biobank
2S	BMI	Summary statistics from GIANT European BMI GWAS *N* = 339 224^48^	Hip OA	Summary statistics from UK Biobank and arcOGEN GWAS of HD hip OA *N* = 15 704 ca, 378 169 co^49^
1S	BMI	Individual-level BMI data in UK Biobank I: BMI SNPs from GIANT^48^	eBMD	Individual-level eBMD data in UK Biobank
2S	BMI	Summary statistics from GIANT European BMI GWAS *N* = 339 224^48^	eBMD	Summary statistics from GEFOS UK Biobank eBMD GWAS *N* = 426 824^46^
1S	Knee OA	Individual-level data on HD knee OA in UK Biobank I: knee OA SNPs from the GO consortium meta-analysis (excluding UK Biobank)^47^	eBMD	Individual-level eBMD data in UK Biobank
2S	Knee OA	Summary statistics from GO consortium GWAS of knee OA, excluding UK Biobank *N* = 44 001 ca, 301 541 co^47^	eBMD	Summary statistics from GEFOS UK Biobank eBMD GWAS *N* = 426 824^46^
1S	Knee OA	Individual-level data on HD knee OA in UK Biobank I: knee OA SNPs from the GO consortium meta-analysis (excluding UK Biobank)	BMI	Individual-level BMI data in UK Biobank
2S	Knee OA	Summary statistics from UK Biobank and arcOGEN GWAS of HD knee OA *N* = 24 955 ca, 378 169 co^49^	BMI	Summary statistics from GIANT European BMI GWAS *N* = 339 224^48^
1S	Hip OA	Individual-level data on HD hip OA in UK Biobank I: hip OA SNPs from the GO consortium meta-analysis (excluding UK Biobank)^47^	eBMD	Individual-level eBMD data in UK Biobank
2S	Hip OA	Summary statistics from GO consortium GWAS of hip OA, excluding UK Biobank *N* = 25 237 ca, 272 284 co^47^	eBMD	Summary statistics from GEFOS UK Biobank eBMD GWAS *N* = 426 824^46^
1S	Hip OA	Individual-level data on HD hip OA in UK Biobank I: hip OA SNPs from the GO consortium meta-analysis (excluding UK Biobank)^47^	BMI	Individual-level BMI data in UK Biobank
2S	Hip OA	Summary statistics from UK Biobank and arcOGEN GWAS of HD hip OA *N* = 15 704 ca, 378 169 co^49^	BMI	Summary statistics from GIANT European BMI GWAS *N* = 339 224^48^

1S, one-sample MR; 2S, two-sample MR; I, instrumented by; ca, cases; co, controls; eBMD, estimated bone mineral density; GEFOS, Genetic Factors for Osteoporosis; GO, Genetics of Osteoarthritis; GWAS, genome-wide association study; BMI, body mass index; HD, hospital-diagnosed; FN–BMD, femoral neck bone mineral density; OA, osteoarthritis; GIANT, Genetic Investigation of Anthropometric Traits; arcOGEN, Arthritis Research UK Osteoarthritis Genetics.

#### One-sample MR

1SMR analyses were performed in the UK Biobank population using the instrumental-variable regression (‘ivreg’) function of the Applied Econometrics with R package.^14^ Exposures were instrumented by an unweighted genetic risk score (GRS), generated as the sum of the dosage for exposure-increasing alleles (data sources provided in Table 1). Analyses were adjusted for age at BMD/BMI assessment, sex, genotyping chip and 40 principal components. Continuous exposures (eBMD/BMI) were standardized before analysis. Effect estimates for binary outcomes (hip/knee OA) were generated from a linear two-stage least-squares regression and represent the increased probability of having OA per unit increase in the exposure. We generated an estimate of the odds ratio (OR) per standard deviation (SD) increase in the exposure, for comparison with 2SMR results, by first regressing the instruments on the exposure, generating predicted values of the exposure, and then regressing the predicted values of the exposure on the binary outcomes using a logistic-regression model. The standard errors for this estimate are likely to be underestimated.^15^

#### Two-sample MR

To maximize the sample size, and thus statistical power, we performed 2SMR using summary-level data from published GWAS. 2SMR analyses were performed using the TwoSampleMR R package, version 0.4.22.^16^ SNP–exposure estimates were extracted for all SNPs associated with the exposure at genome-wide significance. Details of the Genetic Factors for Osteoporosis (GEFOS), Genetic Investigation of Anthropometric Traits (GIANT) and the Genetics of Osteoarthritis (GO) consortium providing the summary statistics for eBMD, BMI and OA, respectively, are provided in the Supplementary Information (available as Supplementary data at *IJE* online). Summary statistics for the eBMD, BMI, hip and knee OA instruments are provided in Supplementary Tables S2–S7 (available as Supplementary data at *IJE* online). Clumping was performed to exclude non-independent SNPs based on a pairwise *r*^2^ > 0.001. SNP–outcome effect estimates were then extracted for independent SNPs. SNP–outcome effect estimates for each analysis are presented in Supplementary Tables S2–S7 (available as Supplementary data at *IJE* online). SNP–exposure and SNP–outcome data were harmonized to ensure that the effect estimates corresponded to the same allele. Palindromic SNPs with indeterminate allele frequencies [minor allele frequency (MAF) > 0.42] were excluded. Steiger filtering excluded SNPs that explained a greater proportion of the variance in the outcome than the exposure.^17^ The proportion of variance explained by each SNP was calculated using the *p*-value and sample size and the ‘get_r_from_pn’ function of the ‘TwoSampleMR’ package for continuous variables and the ‘get_r_from_lor’ function for dichotomous variables. The ‘get_r_from_lor’ function requires the case prevalence in the study population to be specified, which was calculated as the number of cases divided by the total sample size (15% for knee OA and 8% for hip OA). Seven, four and two eBMD SNPs were excluded for analyses with hip OA, knee OA and BMI outcomes, respectively. Two BMI SNPs explained a greater proportion of variance in hip OA risk, 1 for knee OA risk and 15 for eBMD. One knee OA SNP was excluded due to a greater *r*^2^ for eBMD. All Steiger-filtered SNPs are listed in Supplementary Tables S2–S7 (available as Supplementary data at *IJE* online). Estimates were generated using inverse-variance weighted (IVW) random-effects meta-analysis of the Wald ratios for each SNP.

#### Multivariable MR

As we hypothesized that BMI is a confounder of the BMD–OA relationship (i.e. a common causes of both phenotypes), we determined the independent effect of BMD on OA outcomes by performing 1S MVMR including GRS for both BMI and BMD as instruments. Both instruments were regressed on each exposure to generate a predicted value for each exposure. The predicted values for each exposure were then included in a multivariable regression to generate the effect of one exposure on OA when conditioning on the other exposure. Analyses were adjusted for sex, genotyping chip and 40 principal components (PCs). Sanderson–Windmeijer conditional F-statistics were calculated as measures of instrument strength in MVMR analyses.^18^

#### Sensitivity analyses

MR–Egger regression was performed to generate an estimate of horizontal pleiotropy in the two-sample analyses.^19^ Weighted median regression determined the robustness of IVW estimates as weighted median estimates are valid as long as 50% of the information is derived from valid instruments.^20^ We repeated the 2SMR analyses restricted to eBMD SNPs also associated with FN–BMD (*p* < 5 × 10^–8^) in the GEFOS FN–BMD meta-analysis,^21^ to determine whether FN–BMD has a stronger effect than eBMD on hip or knee OA risk. We also performed a latent causal variable (LCV) model, as described by O’Connor and Price,^22^ to determine whether there is a true causal effect of BMD on OA, independently of the genetic correlation. Full methods are described in the Supplementary Information (available as Supplementary data at *IJE* online).

## Results

###  

#### Confirming observational relationships between BMD, OA and BMI in UK Biobank

A total of 334 061 individuals in UK Biobank with genotype data also had measurements of eBMD, BMI, covariates and hospital-diagnosed hip OA; 341 920 had data for knee OA. The mean (SD) age of those with hip OA was 61.7 (6.0), of those with knee OA was 60.2 (6.9) and of controls was 56.2 (8.1) years *(*Supplementary Table S8, available as Supplementary data at *IJE* online). Fifty-seven per cent of people with hip OA were female compared with 50% with knee OA and 54% of the controls. Both hip and knee OA cases were heavier than controls, with mean BMI 28.9 (5.0), 30.3 (5.4) and 27.1 (4.6) kg/m^2^, respectively. Descriptive statistics were virtually the same when restricting to individuals with complete data for eBMD, BMI and OA who were included in the multivariable MR analyses (Supplementary Table S8, available as Supplementary data at *IJE* online).

#### MR analyses provide evidence for bidirectional causal pathways between BMD and OA

**Figure 3:**
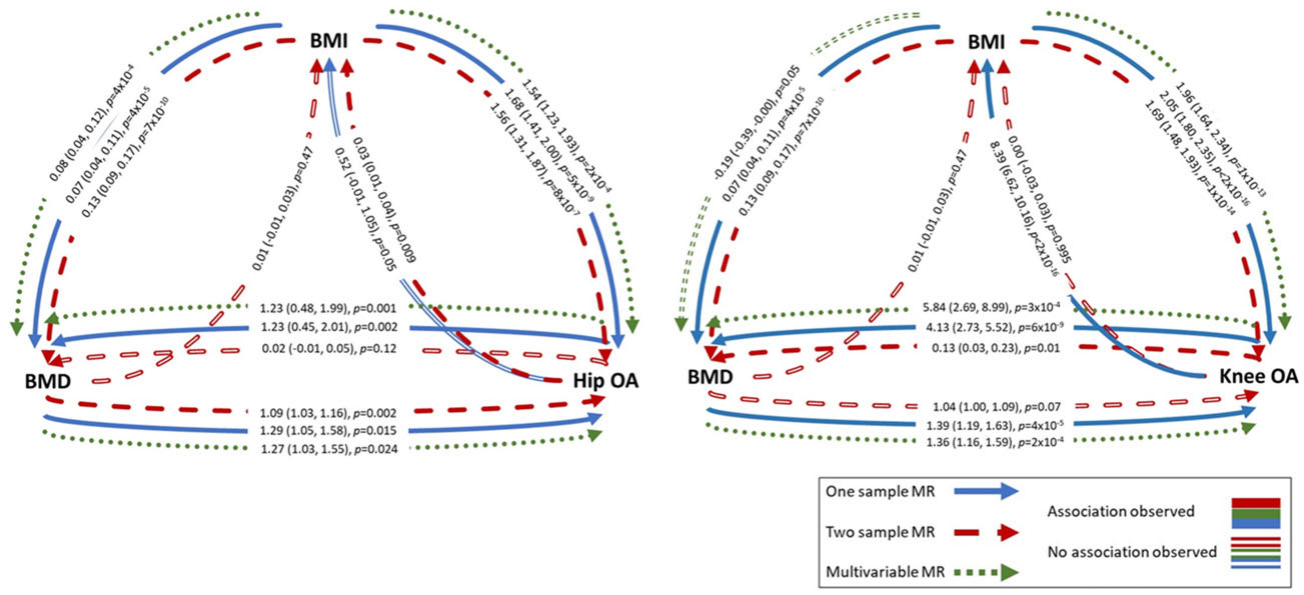
Summary of results of one-sample, two-sample and multivariable Mendelian randomization analyses Effect estimates represent the SD increase in outcome per SD increase in exposure for BMD–BMI and BMI–BMD analyses, the odds ratio per SD increase in exposure for BMI–OA and BMD–OA analyses, and the SD increase in BMD or BMI per 1-unit increase in the log odds of OA. eBMD, estimated bone mineral density; BMI, body mass index; OA, osteoarthritis; MR, Mendelian randomization; SNPs, single-nucleotide polymorphisms.

**Figure 4:**
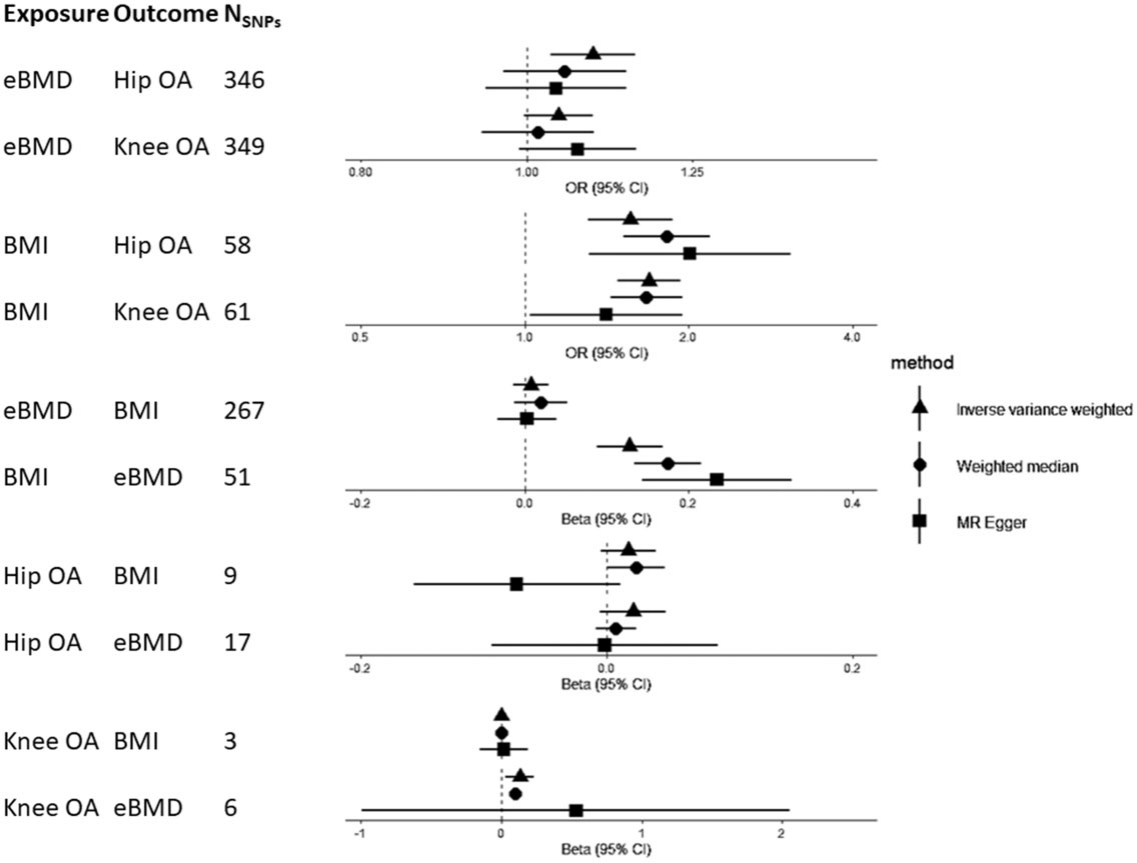
Results of two-sample Mendelian randomization analyses eBMD, estimated bone mineral density; OA, osteoarthritis; BMI, body mass index; CI, confidence interval.

A summary of MR results is presented in Figure 3. In 1SMR, eBMD was causally related to both hip and knee OA, with an SD increase in eBMD related to a 29% [95% confidence interval (CI) = 5, 58] increased odds of having hip OA and 39% (95% CI = 19, 63) increased odds of having knee OA (Table 2). The F-statistic confirmed sufficient instrument strength (F > 3000). Univariable 1SMR results were unaltered by using individual SNPs rather than PRS as instruments, but F-statistics were lower, as was the effect estimate for the causal effect of eBMD on knee OA (Supplementary Table S9, available as Supplementary data at *IJE* online). The BMD risk score was related to BMI but was not related to PA or hormone-replacement-therapy use (Supplementary Table S10, available as Supplementary data at *IJE* online). In 2SMR analyses, IVW provided evidence for a causal effect of eBMD on hip OA [OR per SD increase = 1.09 (95% CI = 1.03, 1.16)], which was relatively consistent (in magnitude) across the three MR methods (Figure 4 and Supplementary Figure S1, available as Supplementary data at *IJE* online). Evidence for a causal effect on knee OA was weaker [OR = 1.04 (95% CI = 1.00, 1.09), Supplementary Figure S2, available as Supplementary data at *IJE* online]. Excluding two SNPs more strongly related to BMI than eBMD did not alter results (Supplementary Table S11, available as Supplementary data at *IJE* online). When restricting to 10 SNPs also associated with FN–BMD (*p* < 5 × 10^–8^) in GEFOS, the magnitude of the effect was stronger for hip OA [OR = 1.40 (95% CI = 1.12, 1.74)], but this effect estimate was less consistent with the MR–Egger and weighted median estimates (Supplementary Table S11, available as Supplementary data at *IJE* online). However, evidence for a causal effect on knee OA was more consistent (in magnitude) across the three methods [OR_IVW_ = 1.21 (95% CI = 1.01, 1.44)].

**Table 2:** Results of one-sample univariable and multivariable Mendelian randomization

	Exposure	Outcome	N_SNPs_ in GRS	F-statistic	*N*	Two-stage least-squares regression		Two-stage linear and logistic regression	
						Estimate (95% CI)	*p-*value	OR (95% CI)	*p*-value
UV MR	eBMD	Hip OA	44	3439	190 181	0.008 (0.001, 0.014)^rd^	0.018	1.29 (1.05, 1.58)	0.015
	eBMD	Knee OA	44	3534	194 410	0.016 (0.008, 0.024)^rd^	4 × 10^–5^	1.39 (1.19, 1.63)	4 × 10^–5^
	BMI	Hip OA	63	4201	333 828	0.016 (0.011, 0.022)^rd^	8 × 10^–9^	1.68 (1.41, 2.00)	5 × 10^–9^
	BMI	Knee OA	63	4446	341 686	0.036 (0.030, 0.043)^rd^	<2 × 10^–16^	2.05 (1.80, 2.35)	<2 × 10^–16^
	BMI	eBMD	63	2953	218 700	0.073 (0.038, 0.108)^sd^	4 × 10^–5^		
	Hip OA	eBMD	10	104	190 181	1.23 (0.48, 1.99)^sd^	0.001		
	Hip OA	BMI	10	201	333 828	0.52 (-0.01, 1.05)^sd^	0.054		
	Knee OA	eBMD	4	50	194 410	4.13 (2.73, 5.52)^sd^	6 × 10^–9^		
	Knee OA	BMI	4	89	341 686	8.39 (6.62, 10.16)^sd^	<2 × 10^–16^		
MV MR	eBMD	Hip OA	44	1795	190 175	0.007 (0.001, 0.013)^rd^	0.028	1.27 (1.03, 1.55)	0.024
	BMI	Hip OA	63	1487	190 175	0.014 (0.006, 0.021)^rd^	3 × 10^–4^	1.54 (1.23, 1.93)	2 × 10^–4^
	eBMD	Knee OA	44	1795	194 404	0.015 (0.007, 0.023)^rd^	2 × 10^–4^	1.36 (1.16, 1.59)	2 × 10^–4^
	BMI	Knee OA	63	1487	194 404	0.034 (0.025, 0.043)^rd^	2 × 10^–13^	1.96 (1.64, 2.34)	1 × 10^–13^
	Hip OA	eBMD	10	66	190 175	1.23 (0.45, 2.01)^sd^	0.002		
	BMI	eBMD	63	1080	190 175	0.080 (0.035, 0.124)^sd^	4 × 10^–4^		
	Knee OA	eBMD	4	53	194 404	5.84 (2.69, 8.99)^sd^	3 × 10^–4^		
	BMI	eBMD	63	865	194 404	–0.193 (–0.387, 3 × 10^–4^)^sd^	0.050		

sd Estimates represent the SD increase in outcome per SD increase in exposure. Estimates for binary exposures represent the SD increase in outcome per doubling in risk of exposure.

rd Estimates for binary outcomes represent the risk difference per SD increase in exposure. Due to the difficult interpretation of these results, odds ratios were also calculated by two-stage regression. Odds ratios are per SD increase in exposure.

MR, Mendelian randomization; BMD, bone mineral density; OA, osteoarthritis; UV, univariable; MV, multivariable; eBMD, estimated BMD.

There was evidence for a causal pathway between hip OA and eBMD in 1SMR [SD increase per doubling in odds of hip OA = 1.23 (95% CI = 0.48, 1.99)] (Table 2), but not 2SMR analysis (Supplementary Figure S3, available as Supplementary data at *IJE* online). Evidence for a causal effect of knee OA on eBMD was provided by 1SMR [β = 4.13 (95% CI = 2.73, 5.52)] and 2SMR [β = 0.13 (95% CI = 0.03, 0.23)], with a positive effect observed for all three 2SMR methods, albeit weaker, with wide CIs overlapping the null for MR–Egger regression (Figure 4 and Supplementary Figure S4, available as Supplementary data at *IJE* online). The knee, but not hip, OA GRS was related to BMI, potentially invalidating instrumental-variable assumption 2 (IV2) (Supplementary Table S10, available as Supplementary data at *IJE* online).

#### BMI is a strong causal risk factor for BMD and OA with weaker evidence for bidirectionality

1SMR provided evidence that BMI has a strong causal effect on hip and knee OA, with an SD increase in BMI associated with a 68% (95% CI = 41, 100) increased odds of hip OA and 105% (95% CI = 80, 135) increased risk of knee OA (Table 2). 2SMR suggested that BMI is causally related to OA, with an SD increase in BMI related to a 56% (95% CI = 31, 87) increased odds of hip OA and a 69% (95% CI = 48, 93) increased odds of knee OA. These results were consistent across the three 2SMR methods (Supplementary Figures S5 and S6, available as Supplementary data at *IJE* online), other than the causal effect of BMI on knee OA estimated by MR–Egger, which was ∼30% weaker, albeit in the same direction (Figure 4). There was strong evidence, from 1SMR, that the causal pathway between BMI and knee OA was bidirectional, with weaker evidence for hip OA (Table 2). Additional adjustment for total weekly PA (assessed using the International Physical Activity Questionnaire) did not attenuate these relationships. 2SMR, however, provided weak and inconsistent evidence (across the three methods) of a causal effect of hip OA on BMI only (Figure 4 and Supplementary Figures S7 and S8, available as Supplementary data at *IJE* online).

We could not perform bidirectional 1SMR for BMD–BMI as the FN–BMD SNPs were identified by weight-adjusted GWAS, meaning the instrument for FN–BMD may be inversely related to weight and thus BMI.^23^ 2SMR using summary statistics from the eBMD GWAS, not adjusted for weight, did not identify a causal effect of eBMD on BMI (Figure 4 and Supplementary Figure S9, available as Supplementary data at *IJE* online). There was robust evidence for a causal effect of BMI on eBMD in 1SMR, with an SD increase in BMI causing a 0.07SD (95% CI = 0.04, 0.11) increase in heel BMD (Table 2). This estimate was like that from 2SMR and the effect size was consistent for IVW, weighted median and MR–Egger analyses, although the MR–Egger intercept did reveal some evidence of horizontal pleiotropy (Figure 4, Supplementary Table S11 and Supplementary Figure S10, available as Supplementary data at *IJE* online).

#### Multivariable MR identifies an independent causal effect of eBMD on OA

Overall, the 1S and 2S analyses provided consistent evidence that BMI is a confounder of the relationship between BMD and hip/knee OA (i.e. a common cause of both phenotypes, Figure 3). We therefore used 1SMVMR to examine the causal effect of eBMD on OA after accounting for BMI. Following adjustment for BMI, eBMD was found to be an independent causal risk factor for both hip and knee OA with a similar magnitude of effect to that observed in MR analyses not accounting for BMI. BMI had a stronger effect than eBMD for both hip and knee OA (Table 2). Sanderson–Windmeijer F-statistics were >1000 for both instruments. Results were generally consistent when using individual SNPs as instruments, although the evidence for a causal effect of eBMD on knee OA was weakened, as was the instrument strength estimated by F-statistics (Supplementary Table S9, available as Supplementary data at *IJE* online).

MVMR provided evidence for a BMI-independent causal effect of OA on eBMD [β_hip_ = 1.23 (95% CI = 0.45, 2.01), β_knee_ = 5.84 (95% CI = 2.69, 8.99), Table 2]. The causal effect of BMI on BMD was independent of hip OA [β = 0.08 (0.04, 0.12)]. When conditioning on knee OA, an inverse effect of BMI on BMD was observed [β = –0.19 (95% CI = –0.39, 0.00)]. This is unlikely to be bias due to conditioning on a common outcome (i.e. collider bias), as genetically predicted OA is not a common outcome.^18^ The BMI-independent causal effect of knee OA on eBMD was not observed when using individual SNPs as instruments (Supplementary Table S9, available as Supplementary data at *IJE* online).

#### LCV analyses provide evidence for a non-pleiotropic causal effect of BMD on OA

To determine whether shared underlying genetic aetiology fully explained the observed causal effect of BMD on OA, we performed LCV modelling using weight-adjusted summary statistics for both FN/LS–BMD and hip/knee OA. The LCV analysis identified evidence for genetic correlations between BMD (measured at both the FN and LS) and OA at both the hip and knee (rho = 0.16–0.23, Supplementary Table S12, available as Supplementary data at *IJE* online). There was evidence for a partial causal effect of BMD at both sites on OA at both sites, independently of genetic correlation and weight, with the largest magnitude of causal effect observed for FN–BMD and knee OA, with a genetic causality proportion of 0.64.

## Discussion

We have found strong evidence for a causal effect of BMD on hip and knee OA using 1SMR, which was relatively consistent with 2SMR. MVMR confirmed that the effect of BMD on OA is independent of BMI. Our results also suggest that there is a bidirectional causal effect between OA and eBMD. We have confirmed strong causal effects of BMI on eBMD, hip and knee OA, with no causal effect of eBMD on BMI. Finally, we have found some evidence of a positive causal effect of knee and hip OA on BMI. The observed causal effect of BMI on eBMD in this adult population is consistent with a previous analysis of a paediatric population (mean age 10 years), in which a causal effect of BMI on FN–BMD was observed.^9^ As seen in this current analysis, Kemp *et al.* found no evidence for a causal effect of BMD on BMI.^9^ The strong causal effect of BMI on both hip and knee OA corroborates previous MR analyses.^5^^,^^24^

The causal effect of eBMD on hip and knee OA that we observed is consistent with previous MR analyses identifying causal effects of FN and LS–BMD on hip and knee OA^5^^,^^24^ and our recent analyses showing that generalized high bone mass (BMD Z-score >3.2 at the hip or L1) is related to greater worsening of osteophyte and joint space narrowing (JSN) severity at both the hip and knee.^25^^,^^26^ Taken together, these findings suggest that bone parameters in general have a causal effect on OA, regardless of the site or method of measurement. However, the magnitude of the effect of eBMD on OA was larger in 2S analyses restricted to SNPs associated with FN–BMD. There are two potential explanations for a stronger effect of BMD on OA when restricting to FN–BMD loci. First, FN–BMD measured by dual-energy X-ray absorptiometry may be a more accurate representation of the biological pathways between bone and cartilage, compared with eBMD, which represents a combination of speed of sound and broadband ultrasound attenuation. Alternatively, since the FN primarily comprises cortical bone, whereas heel BMD is predominantly trabecular,^27^^,^^28^ these findings may reflect the fact that cortical bone is more strongly related to OA pathogenesis compared with trabecular bone. For example, cortical BMD might be expected to correlate more strongly with subchondral plate sclerosis compared with trabecular BMD, which is implicated in the progression of OA.^29^ Inconsistently with the results of our analysis, some previous studies have provided evidence to suggest that high BMD is related to reduced progression of OA,^30^^,^^31^ although this could be explained by index-event bias, in which conditioning on OA leads to spurious associations between OA risk factors.^32^

We have found some evidence for reverse causality in the relationship between eBMD and OA. The positive direction of effect is as expected from artefactual elevation, rather than loss of bone mass due to reduced PA. However, as we do not expect BMD measurements at the heel to be artefactually elevated by features of OA, the observed causal effect of OA on eBMD in 1SMR may reflect biological pleiotropy (i.e. the same underlying biological pathways may be contributing to both phenotypes). Consistently with shared biological mechanisms contributing to both BMD and OA, Hackinger *et al.* identified a genetic correlation between LS–BMD (but not FN) and OA.^11^ By performing a cross-phenotype meta-analysis between OA and LS–BMD, the authors identified a number of known loci, as well as a novel locus in the *SMAD3* gene.^11^ SMAD3 is part of the transforming growth factor β (TGFβ) signalling pathway, which regulates osteoblast differentiation. The first discovered OA loci, growth differentiation factor-5 (*GDF5*), is a ligand for this pathway.^33^ The canonical Wnt signalling pathway is involved in the regulation of osteoblasts and mutations in this pathway can lead to high or low BMD; e.g. activating mutations in low-density lipoprotein receptor-related protein 5 (LRP5, the receptor involved in Wnt signalling activation) cause high BMD.^34^ This signalling pathway has been implicated in OA pathogenesis;^35^ increased levels of a Wnt signalling inhibitor, DKK1, were associated with reduced progression of hip OA in a population of Caucasian women.^36^

However, we did find stronger, more consistent, evidence for an effect of eBMD on OA, as opposed to vice versa. This could reflect the stronger instrument for BMD, but our LCV analyses using the full set of summary statistics provided further evidence for a causal pathway between BMD and OA, not driven by genetic correlation (or confounding by weight as evidenced by the MVMR), suggesting that bone may still have a direct effect on OA, e.g. via increased joint loading or through related structural alterations in the subchondral bone, such as denser subchondral trabecular bone, which has been linked to the progression of JSN.^37^

### Strengths and limitations

We have utilized the largest data sets possible to maximize the power to detect causal effects. We have ensured that there is no overlap between our exposure and outcome populations. We have examined individual-level data in UK Biobank to perform 1SMR to strengthen evidence.

However, we were unable to use eBMD instruments for 1SMR as they were identified in the same population used for analysis; reassuringly, F-statistics suggested that our FN–BMD instrument was of reasonable strength. We did not use the largest available meta-analysis as the source of the BMI instruments due to significant sample overlap with UK Biobank.^38^ However, the Locke *et al.* European-only meta-analysis, which we used for our instrument source for both 1S and 2SMR, still included >300 000 individuals and identified 77 loci; the PRS generated from these SNPs had a strong F-statistic suggesting that the magnitude of effects identified in 1SMR analyses are unlikely to be explained by bias due to weak instruments. Our OA outcomes for 1SMR were based on hospital diagnosis; it is unclear how this phenotype relates to radiographic features of OA, such as JSN, which are commonly used as clinical trial outcomes. Using a severe phenotype as the outcome means reduced power in GWAS and leads to fewer genome-wide significant loci and a greater chance of weak instrument bias (as highlighted by the much smaller F-statistics for the OA instruments). The OA outcomes from the GO consortium included a range of definitions of hip and knee OA, including hospital diagnosis, radiographic evidence and self-reported OA definitions. Heterogeneity in phenotype also reduces the power to detect loci in GWAS. The ORs from 1SMR are estimates and standard errors (SEs) are likely underestimated,^15^ so caution should be taken when interpreting these effect sizes. There may be additional risk factors related to the genetic variants that we did not include in our MVMR models. The UK Biobank population is limited by a latent population structure even after restricting to White Europeans and adjusting for PCs,^39^ which may confound estimates generated by 1SMR. The UK Biobank population examined was White British and all instruments were derived from predominantly White European populations, meaning that we were unable to examine causal effects in non-European populations, limiting generalizability to other ethnicities for whom the prevalence of, and therefore risk factors for, osteoarthritis may differ.^40–43^ The prevalence of OA is higher in men from UK Biobank compared with women, despite evidence in the general population suggesting a higher prevalence of knee OA in women.^44^ This could be explained by selection bias, as women and healthy individuals (i.e. free of OA) are more likely to participate in UK Biobank. Although we adjusted for sex in our analyses, it is possible that there are other variables related to participation in UK Biobank that we could not account for in our analyses. Individuals with OA have a higher risk of premature mortality than the general population,^45^ which could cause further selection bias if those with severe OA are less likely to survive to participate in UK Biobank. However, this selection bias is unlikely to explain the observed *positive* causal effect of OA on BMI, but may explain the *positive* causal effect estimate for OA on eBMD.

## Conclusions

We have found evidence for a BMI-independent causal effect of BMD on hip and knee OA and some evidence for a bidirectional causal effect, which we hypothesize to reflect shared underlying genetic aetiology. We have confirmed strong causal effects of BMI on BMD and hip and knee OA, and have found novel evidence for a causal effect of knee OA on BMI, which did not appear to be mediated by pain-associated reductions in PA. Further analyses are required to determine the shared pathways contributing to both BMD and OA, and to determine the mechanisms by which higher BMD causes OA.

## Notes

The Genetics of Osteoarthritis consortium: Lilja Stefánsdóttir,^1^ Yanfei Zhang,^2^ Rodrigo Coutinho de Almeida,^3^ Tian T. Wu,^4^ Jie Zheng,^5^ Maris Teder-Laving,^6^ Anne-Heidi Skogholt,^7^ Chikashi Terao,^8^ Eleni Zengini,^9^ George Alexiadis,^10^ Andrei Barysenka,^11^ Gyda Bjornsdottir,^1^ Maiken E. Gabrielsen,^7^ Arthur Gilly,^11^ Thorvaldur Ingvarsson,^12,13^ Marianne B. Johnsen,^7,14,15^ Helgi Jonsson,^12,16^ Margreet G. Kloppenburg,^17^ Almut Luetge,^7^ Reedik Mägi,^6^ Massimo Mangino,^18^ Rob R.G.H.H. Nelissen,^19^ Manu Shivakumar,^20^ Julia Steinberg,^11,21,22,23^ Hiroshi Takuwa,^24,25^ Laurent Thomas,^26,7,27,28^ Margo Tuerlings,^1^ George Babis,^29^ Jason Pui Yin Cheung,^30^ Dino Samartzis,^30^ Steve A. Lietman,^31^ P. Eline Slagboom,^3^ Kari Stefansson,^3,12^ André G. Uitterlinden,^32^ Bendik Winsvold,^7,33,34^ John-Anker Zwart,^7,33^ Pak Chung Sham,^35^ Gudmar Thorleifsson,^1^ Tom R. Gaunt,^5^ Andrew P. Morris,^36^ Ana M. Valdes,^37^ Aspasia Tsezou,^38^ Kathryn S.E. Cheah,^39^ Shiro Ikegawa,^24^ Kristian Hveem,^7,40^ Tõnu Esko,^6^ J. Mark Wilkinson,^41^ Ingrid Meulenbelt,^3^ Ming Ta Michael Lee^2,42^ and Unnur Styrkársdóttir^1^

^1^deCODE genetics/Amgen Inc., Reykjavik, Iceland; ^2^Genomic Medicine Institute, Geisinger Health System, Danville, PA 17822, USA; ^3^Department of Biomedical Data Sciences, Section Molecular Epidemiology, Leiden University Medical Center, Leiden, The Netherlands; ^4^Department of Psychiatry, Li Ka Shing Faculty of Medicine, The University of Hong Kong, Hong Kong; ^5^MRC Integrative Epidemiology Unit (IEU), Bristol Medical School, University of Bristol, Oakfield House, Oakfield Grove, Bristol, BS8 2BN, UK; ^6^Estonian Genome Center, Institute of Genomics, University of Tartu, Tartu, Estonia; ^7^K. G. Jebsen Center for Genetic Epidemiology, Department of Public Health and Nursing, Faculty of Medicine and Health Sciences, Norwegian University of Science and Technology, Trondheim, Norway; ^8^Laboratory for Statistical and Translational Genetics, RIKEN Center for Integrative Medical Sciences, Kanagawa, Japan; ^9^5th Psychiatric Department, Dromokaiteio Psychiatric Hospital, Haidari, Athens, Greece; ^10^1st Department of Orthopaedics, KAT General Hospital, Athens, Greece; ^11^Institute of Translational Genomics, Helmholtz Zentrum München, German Research Center for Environmental Health, Neuherberg, Germany; ^12^Faculty of Medicine, University of Iceland, Reykjavik, Iceland; ^13^Department of Orthopedic Surgery, Akureyri Hospital, Akureyri, Iceland; ^14^Research and Communication Unit for Musculoskeletal Health (FORMI), Department of Research, Innovation and Education, Division of Clinical Neuroscience, Oslo University Hospital, Oslo, Norway; ^15^Institute of Clinical Medicine, Faculty of Medicine, University of Oslo, Oslo, Norway; ^16^Department of Medicine, Landspitali The National University Hospital of Iceland, Reykjavik, Iceland; ^17^Department of Rheumatology, Leiden University Medical Center, Leiden, The Netherlands; ^18^Department of Twin Research and Genetic Epidemiology, Kings College London, London, UK; ^19^Department of Orthopaedics, Leiden University Medical Center, Leiden, The Netherlands; ^20^Department of Biostatistics, Epidemiology and Informatics, Perelman School of Medicine, University of Pennsylvania, Philadelphia, PA, USA; ^21^Cancer Research Division, Cancer Council NSW, Sydney, NSW, Australia; ^22^School of Public Health, Faculty of Medicine and Health, The University of Sydney, Australia; ^23^Human Genetics, Wellcome Genome Campus, Wellcome Sanger Institute, Cambridge, UK; ^24^Laboratory for Bone and Joint Diseases, RIKEN Center for Integrative Medical Sciences, Tokyo, Japan; ^25^Department of Orthopedic Surgery, Shimane University, Izumo, Japan; ^26^Department of Clinical and Molecular Medicine, Norwegian University of Science and Technology, Trondheim, Norway; ^27^BioCore—Bioinformatics Core Facility, Norwegian University of Science and Technology, Trondheim, Norway; ^28^Clinic of Laboratory Medicine, St. Olavs Hospital, Trondheim University Hospital, Trondheim, Norway; ^29^2nd Department of Orthopaedics, National and Kapodistrian University of Athens, Medical School, Nea Ionia General Hospital, ‘Konstantopouleio’, Athens, Greece; ^30^Department of Orthopaedics and Traumatology, The University of Hong Kong, Hong Kong; ^31^Musculoskeletal Institute, Geisinger, Danville, USA; ^32^Department of Internal Medicine, Erasmus MC, Medical Center, Rotterdam, The Netherlands; ^33^Department of Research, Innovation and Education, Division of Clinical Neuroscience, Oslo University Hospital, Oslo, Norway; ^34^Department of Neurology, Oslo University Hospital, Oslo, Norway; ^35^Li Ka Shing Faculty of Medicine, The University of Hong Kong, Hong Kong; ^36^Centre for Genetics and Genomics Versus Arthritis, Centre for Musculoskeletal Research, University of Manchester, Manchester, UK; ^37^Faculty of Medicine & Health Sciences, School of Medicine, University of Nottingham, Nottingham, Nottinghamshire, UK; ^38^Laboratory of Cytogenetics and Molecular Genetics, Faculty of Medicine, University of Thessaly, Larissa, Greece; ^39^School of Biomedical Sciences, The University of Hong Kong, Hong Kong; ^40^HUNT Research Center, Department of Public Health and Nursing, Faculty of Medicine and Health Sciences, Norwegian University of Science and Technology, Trondheim, Norway; ^41^Department of Oncology & Metabolism and Healthy Lifespan Institute, University of Sheffield, Sheffield, UK; ^42^Institute of Biomedical Sciences, academia Sinica, Taipei, Taiwan.

## Supplementary data

Supplementary data are available at *IJE* online.

## Ethics approval

UK Biobank received ethical approval from the Research Ethics Committee (REC reference : 11/NW/0382).

## Funding

This work was supported by the Wellcome Trust [grant ref. 20378/Z/16/Z]. C.L.G. was funded by Versus Arthritis [grant ref. 20000]. J.Z. receives salary and start-up funding from the University of Bristol (Vice-Chancellor's fellowship). J.Z. is also supported by the Academy of Medical Sciences (AMS) Springboard Award, the Wellcome Trust, the Government Department of Business, Energy and Industrial Strategy (BEIS), the British Heart Foundation and Diabetes UK [SBF006\1117]. A.H., E.S., R.G., L.P., J.Z., G.D.S., C.L.G. and J.H.T. work in or are affiliated with a University of Bristol and MRC funded unit [MC_UU_00011/1, MC_UU_00011/4].

## Supplementary Material

dyab251_Supplementary_Data

## Data Availability

UK Biobank data are available through a procedure described at https://www.ukbiobank.ac.uk/principles-of-access/. BMD GWAS summary statistics are available to download from the GEFOS website at http://www.gefos.org/?q=content/gefos-data-release. GIANT BMI GWAS summary statistics are available to download at https://portals.broadinstitute.org/collaboration/giant/index.php/GIANT_consortium_data_files#GIANT_Consortium_2012-2015_GWAS_Summary_Statistics. Hip and knee OA GWAS summary statistics from UK Biobank and arcOGEN are publicly available at https://www.ebi.ac.uk/gwas/.
